# Systematic benchmarking of nanopore Q20+ kit in SARS-CoV-2 whole genome sequencing

**DOI:** 10.3389/fmicb.2022.973367

**Published:** 2022-10-13

**Authors:** Junhong Luo, Zixinrong Meng, Xingyu Xu, Lei Wang, Kangchen Zhao, Xiaojuan Zhu, Qiao Qiao, Yiyue Ge, Lingfeng Mao, Lunbiao Cui

**Affiliations:** ^1^School of Public Health, Nanjing Medical University, Nanjing, China; ^2^Hangzhou Baiyi Technology Co., Ltd., Hangzhou, China; ^3^NHC Key Laboratory of Enteric Pathogenic Microbiology, Jiangsu Province Engineering Research Center of Health Emergency, Jiangsu Provincial Center for Disease Control and Prevention, Nanjing, China

**Keywords:** nanopore sequencing, Q20+ kit, SARS-CoV-2, coronavirus, accuracy

## Abstract

Whole genome sequencing provides rapid insight into key information about the Severe Acute Respiratory Syndrome Coronavirus 2 (SARS-CoV-2), such as virus typing and key mutation site, and this information is important for precise prevention, control and tracing of coronavirus disease 2019 (COVID-19) outbreak in conjunction with the epidemiological information of the case. Nanopore sequencing is widely used around the world for its short sample-to-result time, simple experimental operation and long sequencing reads. However, because nanopore sequencing is a relatively new sequencing technology, many researchers still have doubts about its accuracy. The combination of the newly launched nanopore sequencing Q20+ kit (LSK112) and flow cell R10.4 is a qualitative improvement over the accuracy of the previous kits. In this study, we firstly used LSK112 kit with flow cell R10.4 to sequence the SARS-CoV-2 whole genome, and summarized the sequencing results of the combination of LSK112 kit and flow cell R10.4 for the 1200bp amplicons of SARS-CoV-2. We found that the proportion of sequences with an accuracy of more than 99% reached 30.1%, and the average sequence accuracy reached 98.34%, while the results of the original combination of LSK109 kit and flow cell R9.4.1 were 0.61% and 96.52%, respectively. The mutation site analysis showed that it was completely consistent with the final consensus sequence of next generation sequencing (NGS). The results showed that the combination of LSK112 kit and flow cell R10.4 allowed rapid whole-genome sequencing of SARS-CoV-2 without the need for verification of NGS.

## Introduction

Coronavirus disease 2019 (COVID-19), which occurs at the end of 2019, is a very serious infectious disease caused by the severe acute respiratory syndrome coronavirus 2 (SARS-CoV-2) and poses a huge public health challenge to the world (Wu et al., 2020). SARS-CoV-2 is an enveloped virus with a positive-sense, single-stranded RNA genome of ∼30 kb. The COVID-19 epidemic is currently occurring in almost every country in the world, with over 520 million cases of infection and over 6.25 million deaths as of the end of May 2022. Because of the highly transmissible nature of SARS-CoV-2 and the easy mutation nature of single-stranded RNA viruses, SARS-CoV-2 is constantly mutating and undergoing immune escape (Garcia-Beltran et al., 2021; Harvey et al., 2021).

Currently, the world health organization (WHO) has defined five specific Variants of Concern (VOCs^1^), in particular B.1.617.2 (Delta) and B.1.1.529 (Omicron). Delta was the key strain that caused the early COVID-19 epidemic, with the D614G mutation contributing to the rapid spread of SARS-CoV-2 (Korber et al., 2020; Jackson et al., 2021; Plante et al., 2021). Omicron has been responsible for the rapid re-transmission of COVID-19 epidemic since 2021, and the K417N mutation caused the immune escape of Omicron strain against SARS-CoV-2 vaccine (Cao et al., 2021, 2022; Li et al., 2021). In fact, more than 90% of the sites of SARS-CoV-2 genome have been mutated. According to the PANGOLIN SARS-CoV-2 typing system,^2^ hundreds of SARS-CoV-2 genotypes have appeared, and only whole genome sequencing can detect all genotypes at once.

Nanopore sequencing is a technology with many advantages such as simplicity, real-time rapid sequencing, and long reads. It has been used to sequence pathogens in several previous outbreaks, such as Ebola, Zika, and Lassa virus (Hoenen, 2016; Quick et al., 2017; Kafetzopoulou et al., 2019). The earliest artic sequencing protocol for sequencing SARS-CoV-2 was also derived from the nanopore sequencing protocol of the Zika virus (Quick et al., 2017). At present, nanopore sequencing is widely used for the whole genome sequencing of SARS-CoV-2. A large number of SARS-CoV-2 sequences in databases such as Global Initiative of Sharing All Influenza Data (GISAID) and National Center for Biotechnology Information (NCBI) are sequenced by nanopore sequencing. In addition, the nanopore-based direct RNA sequencing is also used to study the subgenomic structure and RNA modification of SARS-CoV-2, providing scientists with the complete transcriptome structure of SARS-CoV-2 (Davidson et al., 2020; Kim et al., 2020; Chang et al., 2021; Wang et al., 2021; Ugolini et al., 2022).

Although nanopore sequencing has excellent performance in SARS-CoV-2 sequencing, with a sensitivity and specificity of more than 99% based on a sequencing depth greater than 60x, compared with the next generation sequencing (NGS) technologies represented by Illumina (Bull et al., 2020). There are still scientists who are concerned about the accuracy of nanopore sequencing and still perform NGS to verify the nanopore sequencing results when studying the transmission relationship between cases. Recently, Oxford Nanopore Technologies (ONT) launched Q20+ kit (LSK112), which claimed to produce duplex data (∼Q30) and achieve simplex accuracies of over 99%, enhanced high-precision consensus sequence as well as mutation identification, when combined with the latest flow cell R10.4. In this study, we firstly utilized Q20+ kit in combination with flow cell R10.4 for whole-genome sequencing of SARS-CoV-2, and we compared the sequencing results with the results of NGS and the combination of the previous nanopore sequencing kit LSK109 and flow cell R9.4.1 to observe whether Q20+ kit showed significant improvement in the accuracy of SARS-CoV-2 whole-genome sequencing. We found that the SARS-CoV-2 consensus sequences of the combination of Q20+ kit and flow cell R10.4 were completely consistent with the sequences generated by the NGS, with a very significant improvement in single-molecule accuracy, particularly for the homopolymer region where nanopore sequencing was most likely to be incorrect in the past. Comparing with the old kit LSK109 with R9.4, the new Q20+ kit (LSK112) with flow cell R10.4 improved the average sequence accuracy in sequencing SARS-CoV-2 96.25% to 98.34% and the proportion of sequences with an accuracy of more than 99 to 30.1% from 0.61%, which greatly reduced the background noise that may interfere with variants calling.

## Materials and methods

### Sample preparation

A total of 15 samples were selected in this study, all of which have been sequenced by NGS, and all of them were provided by the Institutes of Pathogenic Microbiology of Jiangsu Provincial Center for Disease Control and Prevention. The sample information is shown in Table 1. According to the operating instruction of the automatic nucleic acid extractor, RNA was extracted by using accompanying nucleic acid extraction kits. Quantitative reverse-transcriptase polymerase chain reaction (qRT-PCR) of RNA was performed using COVID-19 Coronavirus Real Time PCR Kit (bioPerfectus technologies and Daan Gene, China) in CFX Connect Real-Time PCR Detection System (96-Well 0.2 mL Block) (Bio-Rad, American).

**TABLE 1:** The information of 15 samples.

Strain number	VOCs	Ct value (ORF1ab/N)	Sample type	Source	NGS instrument
20216080-9	Delta	18.8/15.6	Throat Swab	Nanjing	Illumina miseq
20216110-27	Delta	21/24	Throat Swab	Yangzhou	Ion Torrent Genexus
20216085-26	Delta	26/24.5	Throat Swab	Yangzhou	MGISEQ-2000
20216080-10	Delta	23.1/20	Throat Swab	Nanjing	Illumina miseq
20216097-3	Delta	22/21	Throat Swab	Yangzhou	Illumina miseq
20216097-25	Delta	25/23	Throat Swab	Yangzhou	Illumina miseq
20216085-11	Delta	24/25	Throat Swab	Yangzhou	MGISEQ-2000
20216085-30	Delta	25/25	Throat Swab	Yangzhou	MGISEQ-2000, Ion S5 XL
20216085-31	Delta	27/26	Throat Swab	Yangzhou	MGISEQ-2000
2022030-11	Omicron	17/20	Throat Swab	Suzhou	Illumina NextSeq 2000
2022030-8	Omicron	22/23	Throat Swab	Suzhou	Illumina NextSeq 2000
2022030-7	Omicron	18/20	Throat Swab	Suzhou	Illumina NextSeq 2000
2022071-1	Omicron	20/21	Throat Swab	Nantong	MGI DNBSEQ-E5
2022071-2	Omicron	15/16	Throat Swab	Nantong	MGI DNBSEQ-E5
2022071-3	Omicron	14/16	Throat Swab	Nantong	MGI DNBSEQ-E5

### Reverse-transcriptase polymerase chain reaction

Short Fragment (400bp) Target Capture Kit and Long Fragment (1200bp) Target Capture Kit for SARS-CoV-2 Whole Genome (Baiyi Technology Co., Ltd., China, BK-WCoV024TS and BK-WCoV024IITS) were selected to reverse transcribe the extracted RNA and amplify the SARS-CoV-2 whole genome. The top three samples in Table 1 were amplified using the short fragment target capture kit and the other samples were amplified using the long fragment target capture kit. RNA was reverse transcribed into cDNA with reverse transcriptase and random primers, and the cDNA was amplified by multiple polymerase chain reaction (Multiple PCR) using primer pool 1 and primer pool 2 provided in the kit, respectively. The conditions of Multiple PCR: 98°C for 30s followed by 25 cycles of 98°C for 15s, 65°C for 5min, and 72°C for 2min. The Multiplex PCR products were purified by AMPure XP beads (Beckman coulter, United States) and then quantified using a Qubit 2.0 Fluorometer and Qubit dsDNA BR Assay kit (Thermo Fisher Scientific, Q32850).

### Next generation sequencing

Illumina sequencing was performed using Nextera XT DNA Library Preparation Kit (Illumina, FC-131-1096) for library building and sequencing on Miseq or NextSeq 2000 (300 cycles for 150bp paired end read type). BGI sequencing was performed using ATOPlex RNA Library Prep Set for library construction and sequencing on MGISEQ-2000, and using DNBelab-D4RS Digital Sample Preparation System and accompanying kits for library building and sequencing on DNBSEQ-E5. Both sequencers of Applied Biosystems were automatic operating systems, using matching kits and materials for library building and sequencing.

### Nanopore sequencing

Libraries were built according to the manufacturer’s protocols of Sequencing Ligation Kit (ONT, SQK-LSK109 or SQK-LSK112) and Native Barcoding Kit (ONT, EXP-NBD104) or Native Barcoding Kit 24 (ONT, SQK-NBD112.24), respectively. After quantitative dilution, the libraries were loaded onto flow cell R9.4.1 (ONT, FLO-MIN106D) and flow cell R10.4 (ONT, FLO-MIN112), respectively, and were sequenced on GridION X5. The run was terminated after achieving sufficient sequencing data and the flow cell was washed using flow cell Wash Kit (ONT, EXP-WSH004), allowing it to be reused in subsequent sequencings.

### Data analysis

The fast5 electrical signal files were obtained from the nanopore sequencing down-machine data, and then the fast5 data were converted to standard fastq files using Guppy (v 6.0.1^3^) to study the effect of different base-calling strategies on the accuracy of the nanopore sequencing data. We used three modes from the configuration file – config in guppy: dna_r9.4.1_450bps_fast.cfg, dna_r9.4.1_450bps_hac.cfg and dna_r9.4.1_450bps_sup.cfg, corresponding to the conversion modes: fast, hac and sup mode, respectively. The average Q value for each reads was counted using Seqkit tool (v.2.2.0^4^) (Shen et al., 2016) and the accuracy density curves were plotted based on the obtained Q values using the ggplot2 package in R language (v 4.1.3^5^). When analyzing the homopolymer accuracy of the SARS-CoV-2, we used Seqkit tool to obtain all homopolymer positions and corresponding sequences on the reference genome Wuhan-Hu-1, and then used Seqkit tool to count the number of different homopolymers matched to the sample data, using the ggplot2 package for line plotting.

The data analysis process was carried out using BAIYI MicroGeno Platform (v 4.0^6^, Hangzhou Baiyi Technology Co., Ltd.). The raw data were first quality controlled using NanoPlot (v.1.30.0^7^) (Coster et al., 2018) and then the low quality and sequences less than 200bp were filtered using Filtlong (v.0.2.0^8^) based on the quality control results. The filtered clean data were compared with the reference genome Wuhan-Hu-1. When processing the NGS data, we used BWA (v 0.7.17^9^) (Li, 2018) for comparison and minimap2 (v 2.22^10^) (Li, 2018) when processing the nanopore data. Mutation site detection was performed using freebayes (v 1.1.2^11^), with reference assembly of the SARS-CoV-2 whole genome sequence using bcftools (v 1.12^12^) (Danecek et al., 2021). We calculated the Shannon entropy of variant sites in nanopore sequencing and NGS to analyze the accuracy of sequenced sites (formula of Shannon entropy: H(x) = –∑_*x*_ *P*(*x*) *log*_2_[*P* (*x*)]), using the ggplot2 package for line plotting.

## Results

### The basic sequencing data

Fifteen samples were selected for SARS-CoV-2 whole genome sequencing by NGS and nanopore sequencing, including 9 Delta samples and 6 Omicron samples. Short Fragment (400bp) Target Capture Kit and Long Fragment (1200bp) Target Capture Kit for SARS-CoV-2 Whole Genome were selected to reverse transcribe the extracted RNA and amplify the SARS-CoV-2 whole genome. Among nine Delta samples, three samples were amplified by the 400bp capture kit, six samples were amplified by the 1200bp capture kit. Six Omicron samples were amplified by the 1200bp capture kit. Then, fifteen samples were sequenced by NGS, method A (using LSK112 kit with flow cell R10.4) and method B (using LSK109 kit with flow cell R9.4.1) respectively (the details of amplification and sequencing protocol are given in the methods and materials). The time from a sample to sequencing result was 21–29 h for NGS and 7–8 h for nanopore sequencing. Statistical analysis of the sequencing results showed that the sequencing depth of each sequencing method was greater than 230, and the whole genome sequences of 15 samples were basically obtained, with most of sequences coverage above 99% (Table 2). The amount of the sequencing data is showed in Table 3.

**TABLE 2:** Information on whole genome sequencing data from 15 samples.

Strain number	Sequencing depth			Coverage			Average fragment length	
	NGS	LSK109 +R9.4.1	LSK112 +R10.4	NGS	LSK109 +R9.4.1	LSK112 +R10.4	LSK109 +R9.4.1	LSK112 +R10.4
20216080-9	2765	2397	1868	98.90%	99.79%	99.08%	376.155	412.708
20216110-27	16635	1159	545	99.77%	99.78%	99.37%	805.838	881.555
20216085-26	61587	230	434	99.90%	99.61%	99.62%	692.554	801.429
20216085-11	29596	1311	3016	99.90%	99.54%	99.60%	1058.65	1095.62
20216085-30	25627	1669	4137	99.90%	99.53%	99.60%	1064.24	1096.96
20216085-31	39297	1412	3689	99.90%	99.60%	99.61%	1059.76	1091.5
20216080-10	887	3161	873	97.78%	99.60%	98.93%	895.279	518.09
20216097-3	4661	1050	3288	99.69%	99.60%	99.60%	965.401	984.236
20216097-25	1818	2426	3274	99.54%	99.60%	99.60%	992.775	1052.37
2022030-11	19799	1737	5016	99.32%	97.90%	99.28%	1085.94	1111.35
2022030-8	19209	1299	3905	99.34%	96.26%	98.19%	1037.88	1063.16
2022030-7	28698	1615	4823	86.77%	98.29%	98.33%	1083.78	1103.44
2022071-1	3109	7889	3068	99.19%	99.17%	98.60%	1062.55	1082.7
2022071-2	6051	3986	3554	99.54%	98.07%	98.07%	1102.29	1113.51
2022071-3	6231	4565	3899	99.31%	98.61%	98.35%	1084.41	1102.2

**TABLE 3:** The amount of the sequencing data.

Sample	Q20		R9.4	
	Fast	Hac	Fast	Hac
20216080-9	130 Mb	134.9 Mb	104.3 Mb	108.9 Mb
20216110-27	94.8 Mb	100.3 Mb	243.3 Mb	250.6 Mb
20216085-26	105.6 Mb	114.6 Mb	78.1 Mb	80.1 Mb
20216085-11	107.8 Mb	113.8 Mb	48.8 Mb	50 Mb
20216085-30	142.9 Mb	154 Mb	61.3 Mb	62.9 Mb
20216085-31	134.1 Mb	140.3 Mb	52.9 Mb	54.3 Mb
20216080-10	105.8 Mb	111.9 Mb	122 Mb	126.5 Mb
20216097-3	124.8 Mb	128.7 Mb	39.6 Mb	40.9 Mb
20216097-25	119.8 Mb	124.3 Mb	89.4 Mb	92.6 Mb
2022030-11	169.3 Mb	180 Mb	60.7 Mb	62.1 Mb
2022030-8	135.2 Mb	144.4 Mb	47.2 Mb	48.2 Mb
2022030-7	162.7 Mb	172.3 Mb	56.9 Mb	58.2 Mb
2022071-1	106.1 Mb	112 Mb	280.2 Mb	287.6 Mb
2022071-2	120.9 Mb	128.4 Mb	140.9 Mb	144.9 Mb
2022071-3	134.2 Mb	141.9 Mb	161.9 Mb	166.1 Mb

### Analysis of accuracy

#### The effect of different base calling strategies on the accuracy of nanopore sequencing

**FIGURE 1:**
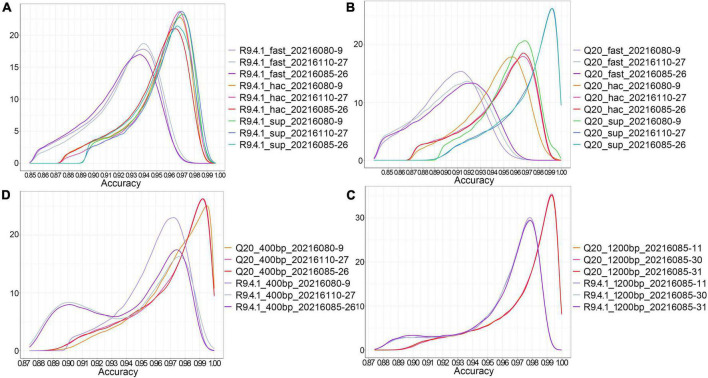
The effect of different base calling strategies on the accuracy of nanopore sequencing. **(A)** The density distribution of sequence accuracy corresponding to three data conversion modes (fast, hac, and sup) in method B; **(B)** The density distribution of sequence accuracy corresponding to three data conversion modes (fast, hac, and sup) in method A; **(C)** The density distribution of sequence accuracy of the 400 bp targeted amplicons sequenced by method A and method B in the sup mode; **(D)** The density distribution of sequence accuracy of the 1200 bp targeted amplicons sequenced by method A and method B in the sup mode.

Considering that nanopore sequencing is a technology based on electrical signal sequencing, different base calling strategies can be chosen during the conversion of electrical signal fast5 data into fastq data. Guppy, providing three base calling strategies (fast, hac, and sup modes), was utilized to analyze the effect of different data conversion modes on sequence accuracy. The density distribution of sequence accuracy showed that the sup mode had higher accuracy for both method A and method B (Figures 1A,B). The fast and hac modes were not suitable for analyzing Q20 data, with the fast mode being more obvious (Figure 1B). Therefore, we consistently chose the sequence accuracy in the sup mode to evaluate both nanopore sequencing methods. It could be found that the sequence accuracy of method A was significantly better than that of method B for both the 400bp capture kit and the 1200bp capture kit. This illustrated that regardless of the length of the sequenced fragments, Q20+ kit had a great improvement in sequence accuracy, reaching an accuracy of 99% (Figures 1C,D).

#### The effect of different amplicon lengths on the accuracy of nanopore sequencing

**FIGURE 2:**
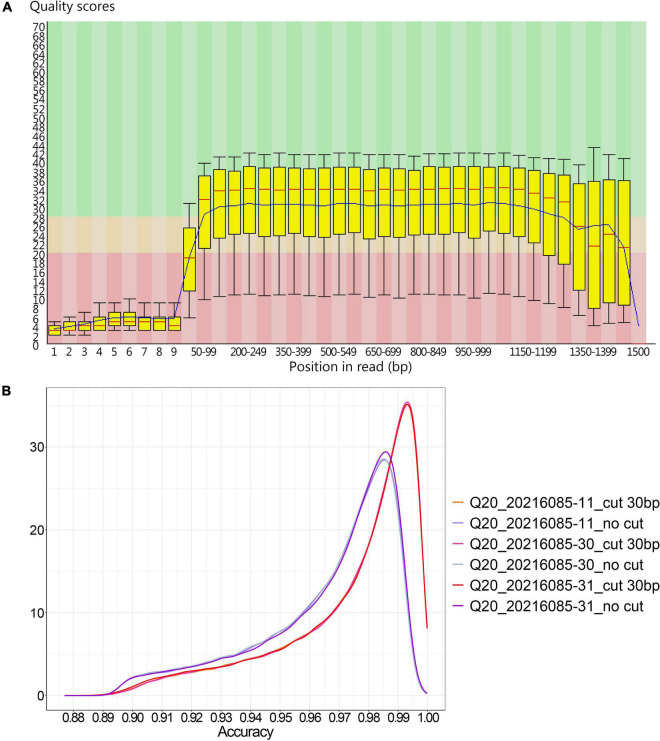
The accuracy of 1200 bp amplicons sequenced by method A. **(A)** The single-base Q value corresponding to the base position in the 1200 bp amplified product from method A; **(B)** The density distribution of sequence accuracy of the 1200 bp amplified product with and without the 30 bp adapter sequence cut in method A.

In method B, the average sequencing fragment lengths obtained by using the 400bp capture kit and the 1200bp capture kit were around 376bp and 1058bp, respectively, with no significant difference in accuracy. In contrast, in method A, the average reads accuracy of the 1200bp amplicon improved significantly compared to that of the 400bp amplicon, from 96.5 to 97.5%, and the average proportion of data above Q20 rose from 23 to 28.8% (Table 4). This led us to further consider whether different amplicon lengths had an effect on nanopore sequencing accuracy? As could be seen in the single-base accuracy analysis, the average single-base Quality value (Q value) of the 400bp amplicon was indeed lower than that of the 1200bp amplicon (Supplementary Figure 1). Interestingly, we also found that in method A, the single-base Q values for the first 20-30 bp was very low (Figure 2A), possibly due to an unstable electrical signal generated when the DNA fragment just passed through the nanopore. However, the first 20–30 bp was the adapter sequence, not the true amplified fragment sequence. With this in mind, we further performed statistics on the accuracy after cutting the adapter sequence and found that was 98.27% (Figure 2B).

**TABLE 4:** The accuracy of whole genome sequencing of 15 samples.

Strain number	Targeted capture fragment length	Sequence accuracy			De-adapter sequence accuracy		Q20+ data	
		NGS	LSK109 +R9.4.1	LSK112 +R10.4	LSK109 +R9.4.1	LSK112 +R10.4	LSK109 +R9.4.1	LSK112 +R10.4
20216080-9	400bp	99.98%	95.32%	96.02%	96.37%	98.14%	1.14%	23.76%
20216110-27	400bp	99.68%	95.32%	96.61%	95.73%	98.00%	0.57%	23.76%
20216085-26	400bp	99.75%	95.43%	96.53%	96.11%	98.00%	1.43%	23.51%
20216085-11	1200bp	99.70%	96.28%	97.25%	96.69%	98.26%	0.84%	29.16%
20216085-30	1200bp	99.68%	96.20%	97.25%	96.69%	98.30%	0.86%	29.35%
20216085-31	1200bp	99.72%	96.20%	97.31%	96.69%	98.26%	0.88%	29.04%
20216080-10	1200bp	99.98%	95.43%	97.37%	96.20%	98.30%	0.47%	27.08%
20216097-3	1200bp	99.98%	95.53%	96.45%	96.20%	98.71%	0.27%	38.12%
20216097-25	1200bp	99.97%	95.53%	97.66%	96.20%	98.59%	0.31%	33.56%
2022030-11	1200bp	99.94%	96.37%	97.60%	96.76%	98.30%	0.67%	29.60%
2022030-8	1200bp	99.94%	96.37%	97.30%	96.70%	98.20%	0.86%	28.60%
2022030-7	1200bp	99.94%	96.37%	97.30%	96.70%	98.30%	0.78%	30.10%
2022071-1	1200bp	99.99%	96.02%	97.30%	96.40%	98.20%	0.48%	28.50%
2022071-2	1200bp	99.99%	96.02%	97.30%	96.30%	98.20%	0.44%	28.80%
2022071-3	1200bp	99.99%	96.02%	97.30%	96.40%	98.20%	0.46%	29.20%

#### The effect of duplex data on the accuracy of nanopore sequencing

**FIGURE 3:**
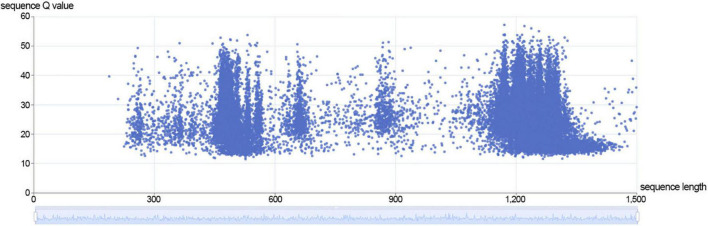
The distribution of sequence length and sequence Q value of duplex data in method A.

Currently, for DNA sequencing, ONT only supports the 1D method, but LSK112 kit is supported by the 2D method. Compared to method B, some sequences in method A are double stranded through the nanopore. In the sequences with positive and negative strand through the nanopores, we used Guppy (guppy_basecaller_duplex) with duplex tools (v 0.2.9^13^) for method A to analyze the extracted duplex data. The statistical analysis revealed an average Q value of 26.1 for the duplex data, corresponding to an accuracy of 99.75453%, and duplex data accounted 3.33% of the sequencing data of method A (Figure 3), which was relatively in line with the 1-10% range given by ONT. The results showed that duplex data was particularly effective in improving the accuracy of nanopore sequencing.

### Analysis of single nucleotide polymorphism and insertion-deletion

Taking the SARS-CoV-2 genome Wuhan-Hu-1 (GenBank accession number: MN908947.3) as the reference genome, we analyzed the mutation site for each sample. It could be found that method B had a significant increasement of mutation sites in the fast mode, 30.97% of which were caused by homopolymer variation, and generated 2.92% false positive site heterozygosity in addition (Supplementary Table 1). This also confirmed that the fast mode was not suitable for accurate variants calling, meanwhile the fast mode can run faster than sup mode with lower hardware requirement, which may illustrate why some scientists still have doubt about the accuracy of nanopore sequencing technology even with the rapid development of accuracy in nanopore sequencing. In the sup mode, method A and method B were completely consistent with the NGS in mutation detection with the consistent site coverage. Intriguingly, in method B, we analyzed eight consecutive T-base position (genomic position 11094) in the sup mode, and found that 7 out of 15 samples were identified to be heterozygous with a deletion of one T base which proportion was greater than 50%, and the other 8 samples had low heterozygosity deletion variation. This false positive situation was well resolved by method A in the sup mode, with none of the 15 samples generating false positive at this position.

### Analysis of homopolymer

**FIGURE 4:**
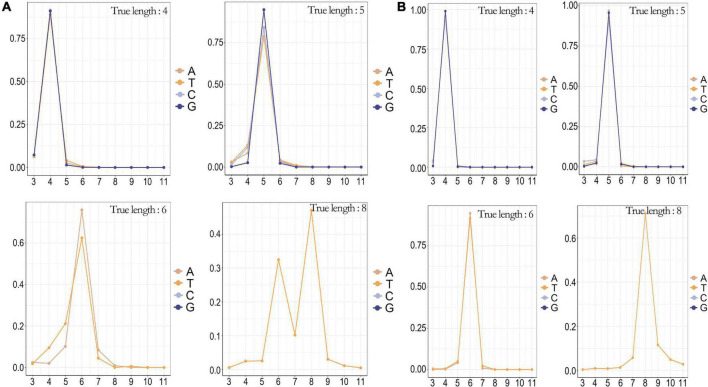
The counterr density statistic for the detection of different homopolymers on the whole genome of SARS-CoV-2. **(A)** The counterr density plot of method B in the sup mode for the detection of homopolymer with lengths of 4, 5, 6, and 8; **(B)** The counterr density plot of method A in the sup mode for the detection of homopolymer with lengths of 4, 5, 6, and 8.

We conducted a genome-wide scan of the SARS-CoV-2 whole genome, which had multiple regions of homopolymer, including a T-base homopolymerized region with a length up to 8, in addition to the 3′ UTR. In method B, the percentage of homopolymer identification accuracy gradually decreased as the length of homopolymer increased (Figure 4A). This limited the application of this sequencing method to whole-genome sequencing of SARS-CoV-2, as it could easily cause frame shift mutation. Method A showed high recognition accuracy for homopolymer, and still had excellent recognition accuracy for a T-base homopolymer region with a length of 8 (Figure 4B). Moreover, the recognition accuracy of homopolymer was significantly negatively correlated with the length of homopolymer, and had no significant correlation with the four base types.

### Analysis of data quantity

**FIGURE 5:**
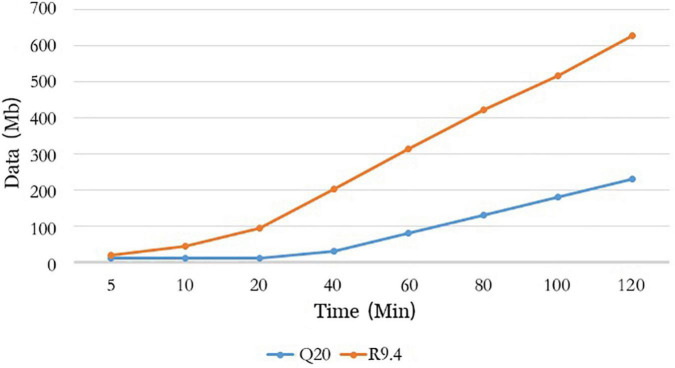
Comparison of method A with method B for data output over time.

We analyzed the data quantity generated by flow cell R10.4 and flow cell R9.4.1 over time and could see that flow cell R10.4 generated approximately 230 Mb data at 120 min, which is a significant difference compared to the 625.4 Mb data generated by flow cell R9.4.1 (Figure 5). It was a significant positive correlation with the speed through the nanopore of sequences on both flow cells. The sequencing speed of flow cell R9.4.1 is 400∼450bp per second, while the sequencing speed of flow cell R10.4 is reduced to 200bp per second. As could be seen from the above analysis, method A significantly improved sequencing accuracy at the sacrifice of its data output. However, during the whole genome sequencing of SARS-CoV-2, the data output was often excessive, so the combination of LSK112 kit and flow cell R10.4 could still meet the needs of the whole genome sequencing of SARS-CoV-2.

## Discussion

Whole-genome sequencing is the best way to detect SARS-CoV-2 due to its rapidly mutating nature. On account of the advantages of rapid, simple and low-cost sequencing nature, nanopore sequencing technologies is widely used to obtain the whole genome sequence of viruses, such as Ebola, Zika, and Lassa viruses (Hoenen, 2016; Quick et al., 2017; Kafetzopoulou et al., 2019). Actually, how to make the accuracy of nanopore sequencing technology comparable to NGS or even sanger sequencing is still the most important issue to the users. Excitingly, the emergency of nanopore Q20+ kit (LSK112 kit with flow cell R10.4) may help us to sequence the SARS-CoV-2 genome without verification from NGS or sanger sequencing, and its sequencing accuracy has been verified in bacterial, fungal, human and plants (Sereika et al., 2021; Keraite et al., 2022; Sanderson et al., 2022). This study is the first benchmark test of nanopore Q20+ sequencing in SARS-CoV-2 and viruses. Excitingly, LSK114 kit with flow cell R10.4.1 released in London Calling 2022 not only maintains the accuracy of 99%, but also improves the sequencing yield to the same level or even more as LSK109 kit with flow cell R9.4.1.

Regardless of method A or method B, there were significant differences in accuracy among three base calling modes, with the sequence accuracy decreasing significantly in the fast mode, especially in the homopolymer region. Highest accuracy was achieved by two sequencing methods in the sup mode, with some sample sequences in method A reaching an accuracy of over 99%. Method A was more accurate than method B regardless of the size of the targeted capture fragment. And the longer the fragment, the more accurate it was. Method A had duplex data with an average Q value of 26.1 and an accuracy of 99.75453%, although the percentage of duplex data was small. With the development of nanopore sequencing technology and the increasing proportion of duplex data, nanopore sequencing is expected to achieve even higher accuracy. It could be observed that LSK112 kit did improve sequencing accuracy compared to LSK109 kit and was more suitable for sequencing long amplicons. The sequencing quality of sequences that initially enter the nanopore is poor, due to the unstable speed of the initial sequence through the nanopore. The overall sequence accuracy is greatly affected when the length of amplicon was short. It could be seen from the results that the accuracy was significantly improved after removing the adapter sequence. Therefore, we need to filter the adapter sequence and short fragment in order to achieve better analysis results in the data processing part.

In the mutation detection, it was evident that method B had a recognition error in the homopolymer region, which led to the eventual problem of frame shift mutation. This problem is even more noticeable on ONT MK1C platform and this weak point was eliminated on ONT GridION platform supporting the sup base calling mode with a huge boost of read-time computing power. Q20+ kit maintained high recognition accuracy in the homopolymer regions of the lengths of 4, 5, 6 and 8, which clearly showed that the Q20+ kit solved the homopolymer accuracy problem well. We compared the consensus sequences sequenced by method A with the consensus sequences from NGS, and the sequences were identical. The homopolymer region has been a high-incidence region with accuracy problems in the previous sequencing kits of nanopore sequencing technology. However, the continuous upgrades of sequencing kits, flow cell and algorithm are solving the shortcoming, especially Q20+ kits, such as LSK112 kit and LSK114 kit, have improved the ability of detecting homopolymers up to length of 10∼12. A recent study reported sequencing bacteria genome with LSK112 kit and flow cell R10.4 has allowed high accuracy in homopolymers regions of length up to 9 (Sereika et al., 2021). It means that LSK112 kit and flow cell R10.4 allow the accurate detection of the largest 8-base homopolymer in SARS-CoV-2 genome.

In conclusion, Q20+ kit was found to be more accurate than previous nanopore sequencing kits, especially for sequencing long amplicons. The improvement in accuracy derived from the increased 5 to 10% of duplex data, and the relatively reduced sequencing speed that resulted in increased homopolymer identification accuracy. However, to ensure high accuracy, the base calling strategy required selecting the sup mode.

At present, Nanopore sequencing is increasingly used for the whole genome sequencing of SARS-CoV-2 due to its advantages of simple, fast and real-time sequencing. The improved accuracy brought by Q20+ kit can play a more accurate and positive role in the prevention and control of epidemics and traceability analysis of SARS-CoV-2.

## Data availability statement

The data presented in this study have been submitted to the National Genomics Data Center (https://ngdc.cncb.ac.cn/) with submission number: CRA007743. The generated consensus sequences were submitted with accession numbers: GWHBJYX01000000–GWHBKAG01000000.

## Author contributions

JL and XX: methodology establishment, data sorting and analysis, visualization, and writing – original draft. ZM: methodology establishment, data sorting, and writing – review and editing. LW: data sorting and analysis. KZ, XZ, QQ, and YG: resource and writing – review and editing. LM: conceptualization, project administration, supervision, and writing – review and editing. LC: conceptualization, funding acquisition, project administration, validation, supervision, and writing – review and editing. All authors contributed to the article and approved the submitted version.

## Footnotes

1
https://www.who.int/en/activities/tracking-SARS-CoV-2-variants/

2
https://cov-lineages.org/lineage_list.html

3
https://community.nanoporetech.com/docs/prepare/library_prep_protocols/Guppy-protocol/v/gpb_2003_v1_revai_14dec2018

4
https://github.com/shenwei356/seqkit

5
https://www.r-project.org/

6
http://www.baiyi-tech.cn/

7
https://github.com/wdecoster/NanoPlot

8
https://github.com/rrwick/Filtlong

9
https://github.com/lh3/bwa

10
https://github.com/lh3/minimap2

11
https://github.com/freebayes/freebayes

12
https://github.com/samtools/bcftools

13
https://github.com/nanoporetech/duplex-tools/
