# Corrigendum: Systematic benchmarking of nanopore Q20+ kit in SARS-CoV-2 whole genome sequencing

**DOI:** 10.3389/fmicb.2022.1095739

**Published:** 2022-12-15

**Authors:** Junhong Luo, Zixinrong Meng, Xingyu Xu, Lei Wang, Kangchen Zhao, Xiaojuan Zhu, Qiao Qiao, Yiyue Ge, Lingfeng Mao, Lunbiao Cui

**Affiliations:** ^1^School of Public Health, Nanjing Medical University, Nanjing, China; ^2^Hangzhou Baiyi Technology Co., Ltd., Hangzhou, China; ^3^NHC Key Laboratory of Enteric Pathogenic Microbiology, Jiangsu Province Engineering Research Center of Health Emergency, Jiangsu Provincial Center for Disease Control and Prevention, Nanjing, China

**Keywords:** nanopore sequencing, Q20+ kit, SARS-CoV-2, coronavirus, accuracy

In the published article, there was a mistake in the **Funding** statement. The grant number for the Natural Science Foundation of Jiangsu Province was displayed as “BK2021131.” The original **Funding** statement was written as:

“This study was supported in part by the Key Research and Development Project of Jiangsu Province (BE2019761), Natural Science Foundation of Jiangsu Province (BK2021131), and Key Scientific Research Project of Jiangsu Provincial Health Commission (ZD2021060).”

The correct **Funding** statement appears below:

