# Supplementary figures and images for: Systematic benchmarking of nanopore Q20+ kit in SARS-CoV-2 whole genome sequencing
Source: Front Microbiol. 2022 Oct 13;13:973367. doi: 10.3389/fmicb.2022.973367 (PMC9612837; doi:10.3389/fmicb.2022.973367)

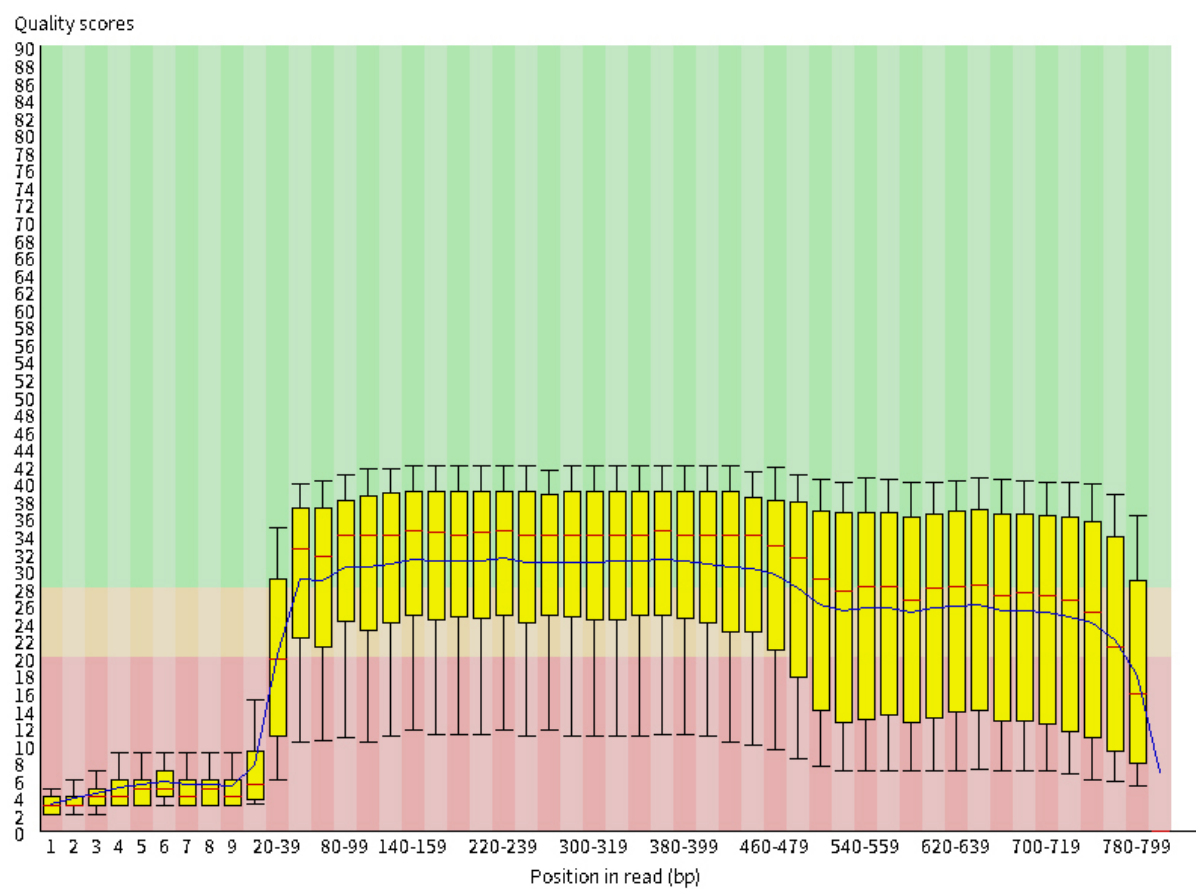

Figure S1. The accuracy of 400bp amplicons sequenced by method A.

Supplement: Supplementary file 1 [file Image_1.pdf]
